# *Candida albicans* Infections: a novel porcine wound model to evaluate treatment efficacy

**DOI:** 10.1186/s12866-022-02460-x

**Published:** 2022-02-04

**Authors:** Joel Gil, Michael Solis, Alexander Higa, Stephen C. Davis

**Affiliations:** Present Address: Miller School of Medicine, Dr. Phillip Frost Department of Dermatology and Cutaneous Surgery Wound Healing Research Laboratory Miami, University of Miami, Miami, 33136 FL United States

**Keywords:** Candida albicans, Antifungal, Porcine, Wound, Biofilm model, New model, Yeast, Porcine model, Infection, Burns, Antifungal

## Abstract

*Candida albicans* is a common cause of opportunistic mycoses worldwide and a major contributor in wound infections. The purpose of this study was to establish a fungal wound model and analyze the effects of a common antifungal agent against the proliferation of three *C. albicans* strains. Second degree burns were created, and then inoculated with one of three different *C. albicans* ATCC strains: 10261 reference strain, 64550 fluconazole resistant and 26310 fluconazole sensitive. After fungal inoculation, every wound was covered with dressings for 4 h to allow fungal colonization on every wound bed. After 4 h, the dressings were removed, and each wound was treated either once or twice daily with a topical terbinafine hydrochloride or left untreated. On days 2, 4 and 7 post inoculation, three wounds from each treatment group were scrub cultured and quantified. On day 2, wounds infected with the sensitive strains 26310 and 10261 and treated twice showed a significant reduction when compared against those infected wounds receiving once daily treatment. On day 4, wounds which were infected with *C. albicans* fluconazole sensitive (ATCC 26310) showed a significant reduction in fungal cell counts with treatment applied twice daily. A significant reduction in the colony counts was exhibited in all three strains at the seventh day with active as compared to the non-treated wounds. Twice daily treatment resulted in a lower fungal count than once daily treatment. Neither treatment was able to entirely eradicate *C. albicans* during the duration of this study. Establishing a reliable fungal wound model will help in the translational goal of identifying new antifungal that could be used clinically by wound care providers.

## Introduction

Superficial fungal infections, such as dermatophytosis, onychomycosis and superficial *Candida* infections, are common and can be caused by a wide range of fungi [1]. Fungal infections are a common cause of morbidity, mortality, and cost in critical care populations, including burns [1–5]. Also fungus infection as Candidiasis has been found in chronic and surgical wounds [6, 7].

*C. albicans* is a normal component of the gastrointestinal tract, the oral cavity, and the vagina. It’s also an opportunistic pathogen, commonly causing infections such as denture stomatitis, thrush, burn infections and urinary tract infections. *C. albicans* can also cause more serious systemic infections, these infections are often transmitted in hospitals [8].

Thermal injury is a serious type of trauma requiring care in specialized units. It is estimated that about 2.5 million individuals in the United States sustain burns requiring medical attention each year [9]. More than 100,000 of these patients are hospitalized, and there are approximately 12,000 deaths per year due to thermal injury [10]. Burn patients are cited as being among the highest risk groups for invasive fungal infections [11–16]. The burn patients loss the barrier function of the skin [17, 18], the use of topical and systemic antibiotics to control bacterial infections [8, 19, 20], and the alteration of the immune system [21–23] leaves the thermally injured patient at an increased risk of infection by opportunistic organisms, including *Candida*. Several species of *Candida* have become common secondary pathogens that have become responsible for a growing number of deaths in burn patients [5, 6, 8, 17, 24–26].

The list of antifungals used to treat infections with *Candida* is extensive. Some of these treatments include topical treatments such as chlorohexidine, clotrimazole, miconazole and ketoconazole; and oral therapies like amphotericin B, nystatin and itraconazole [27, 28]. The efficacy of each treatment depends of the specific virulence of each *Candida* species and the area of the infection [29]. Terbinafine has been demonstrated to have antifungal activity *in vitro* [30–32]. Widely varying minimum inhibitory concentration (MIC) have been reported for *Candida* species, and terbinafine has generally been considered to have limited activity against *Candida albicans* yeasts *in vitro* [33]. In several *in vitro* studies terbinafine activity against *C. albicans* has been primarily fungistatic [34, 35]. The mechanism of action of terbinafine involves the specific inhibition of fungal squalene epoxidase, resulting in ergosterol deficiency and accumulation of intracellular squalene that interfere with normal fungal membrane function in *C. albicans* [36–38].

While other animal models have been used to test the efficacy of antifungals, the majority of these use rats, mice and guinea pigs [39–44]. Since swine have skin that is anatomically and physiologically similar to humans and a strong correlation in wound healing studies have been seen, we used them as our experimental animal [45–50]. The purpose of this study was to describe a porcine burn model for the study of *C. albicans* wound infections and determine if over-the-counter (OTC) treatments are effective against different antibiotic resistant strains. This porcine burn model has been used previously to evaluate several agents *in vivo* on bacterial infections [51–53]. In this model we studied three (3) different *C. albicans* strains including a commonly used strain, a fluconazole resistant and a fluconazole sensitive strains [54, 55]. The selected strains of American Type Culture Collection (ATCC) were selected to effectively compare the treatment groups in this model. *C. albicans* ATCC 10261 is a commonly used reference strain to challenge antifungal agents [56–58]. *C. albicans* ATCC 64550 has been used as a fluconazole-resistant Candida strain in previous studies [59, 60]. *C. albicans* ATCC 26310 utilized as a fluconazole-sensitive strain in several studies [61–63]. This study also compared once versus twice topical terbinafine hydrochloride treatment regimens to determine if the frequency of treatment application made any differences in antifungal activity.

## Methods and materials

### Experimental animals

Two young female specific pathogen free (SPF) pigs (*Sus scrofa domesticus*) were purchased from Looper Farm (Granite Falls, NC) weighing 25–30 kg were kept in house for two weeks prior to initiating the experiment. These animals were fed a basal diet *ad libitum* and housed individually in our animal facilities (meeting USDA compliance) with controlled temperature (19–21 °C) and lights (12 h/12 h LD).

### Anesthetics, analgesics and euthanasia

The animals were anesthetized with a cocktail dose (intramuscular injection) prepared with their corresponding weights. The cocktail used for sedation was made with Telazol HCl (100 mg/mL), given at a dose of 1.4 mg per kilogram; Xylazine (100 mg/mL), given at a dose of 2.0 mg per kilogram; and Atropine (0.54 mg/mL) given at a dose of 0.05 mg per kilogram. Once sedated, animals had endotracheal tube inhalation of an isoflurane and oxygen combination during each procedure. For analgesics, each animal received a fentanyl transdermal patch (50 µg per hour) and Buprenorphine (0.3 mg/mL), given at a dose of 0.03 mg per kilogram. Upon the completion of the wound recoveries, the animals were first anesthetized and after the procedure was finished each animal by euthanized by receiving via an intramuscular injection Euthasol (pentobarbital sodium 390 mg/mL), given at a dose of 1 mL per 10 lbs.

### Wounding

Prior to surgery the animals were anesthetized, the hair on the back of the pigs was clipped with standard animal clippers. Skin on both sides of the animals was prepared by washing with a non-antibiotic soap (Neutrogena®) and sterile water. The animals were blotted dry with sterile gauze. Eighty one (81) second-degree burn wounds were made in the paravertebral and thoracic area on each animal by using specially designed cylindrical brass rods weighing 358 g that was heated in a boiling water bath to 100 °C [64]. A rod was removed from the water bath and wiped dry before it is applied to the skin surface to prevent water droplets from creating a steam burn on the skin. The brass rod was held at a vertical position on the skin (six seconds), with all pressure supplied by gravity, to make a burn wound 8.5 mm diameter × 0.8 mm deep. Immediately after burning, the roof of the burn blister was removed with a sterile spatula. Eighty-one burns were created on each animal for a total of one hundred sixty-two wounds.

### Experimental Design

**Fig. 1:**
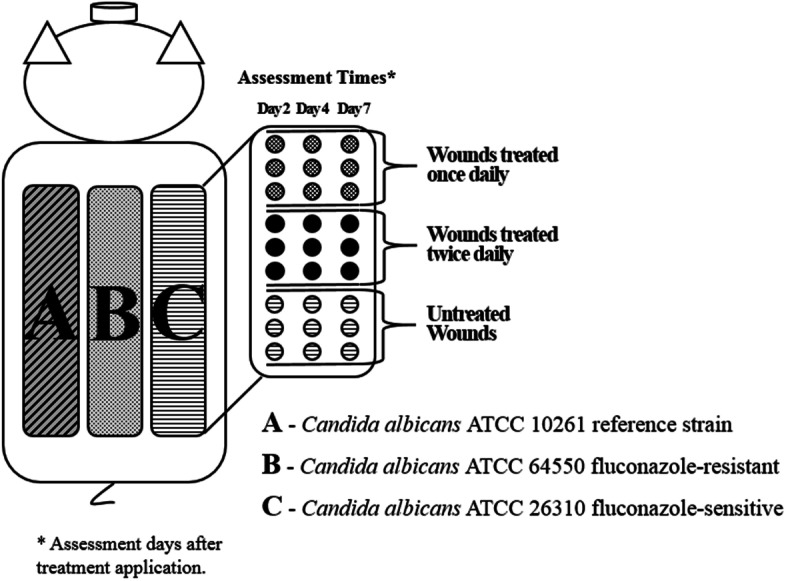
Experimental Design

On each animal, twenty-seven (27) burn wounds were assigned to one of three *C. albicans* strains. (Fig. 1: strains below). Nine burns were allocated for three treatment regimens (described below). The burn wounds were made approximately 3–5 cm from each other. Groups of burns were inoculated, treated, and recovered as described below.

### Inoculation

Fresh cultures of pathogenic isolates were obtained directly from American Type Culture Collection (ATCC, Rockville, Maryland). The inoculums were *Candida albicans* ATCC 10261 (reference strain), *Candida albicans* ATCC 64550 fluconazole-resistant and *Candida albicans* ATCC 26310 fluconazole-sensitive. The frozen fungus was recovered from glycerol stock (15% glycerol, -80 °C). All inoculums’ suspensions were made by scraping the overnight growth from a culture plate into 5 ml of normal saline. This resulted in a suspension concentration of approximately 10 [8] colony forming units/ml (CFU/ml). A small amount of the inoculum suspension was plated onto culture media BBL™ CHROMagar™ Candida Medium (Becton Dickinson GmbH, Heidelberg/Germany) to quantify the exact amount of viable organisms. The inoculum suspension was used directly to inoculate each site. A 0.025 ml (25 μl) aliquot of the suspension was deposited into the center of each burn. The suspension was lightly scrubbed into the test site for ten seconds using a sterile Teflon spatula and left for 3 min prior to covering with a polyurethane film dressing (Tegaderm Transparent Dressing; 3 M Health Care, St. Paul, MN USA) for 4 h to allow the organism colonized the burn area. The film dressings were secured in place with tape and the animals were wrapped with self-adherent bandages.

Four hours post-fungal inoculation, wounds were uncovered and treated with antifungal agent (*topical terbinafine hydrochloride*). Wounds remained either untreated or received either once or twice daily treatment for six days. Inoculated wounds were randomized around the animal within their designated group. Approximately 250 µl of the treatment was applied over each wound and then gently spread over the wound and adjacent normal skin with a sterile applicator. Treatments including untreated control were covered with same dressing as previously described to prevent any cross contamination of treatments.

### Microbiological recovery

Nine burns were cultured from each group at Days 2, 4 and 7 post inoculation from each *C. albicans* strain. Each burn was cultured only once. The area was encompassed by a sterile stainless-steel cylinder (22 mm outside diameter) held in place by two handles. One ml of scrub solution was pipetted into the stainless-steel cylinder and the site was scrubbed with a sterile Teflon spatula for 30 s using a modified scrub technique [65]. Serial dilutions of all recoveries were made and recovered bacteria was quantified using the Spiral Plater System, which deposits a small-defined amount (50 µl) of suspension over the surface of a rotating agar plate. *Candida albicans* was grown on selective BBL™ CHROMagar™ Candida Medium (Becton Dickinson GmbH, Heidelberg/Germany) overnight at 37 °C. Colonies on the plates were counted and the colony forming units per mL (CFU/ml) calculated.

## Results

**Fig. 2:**
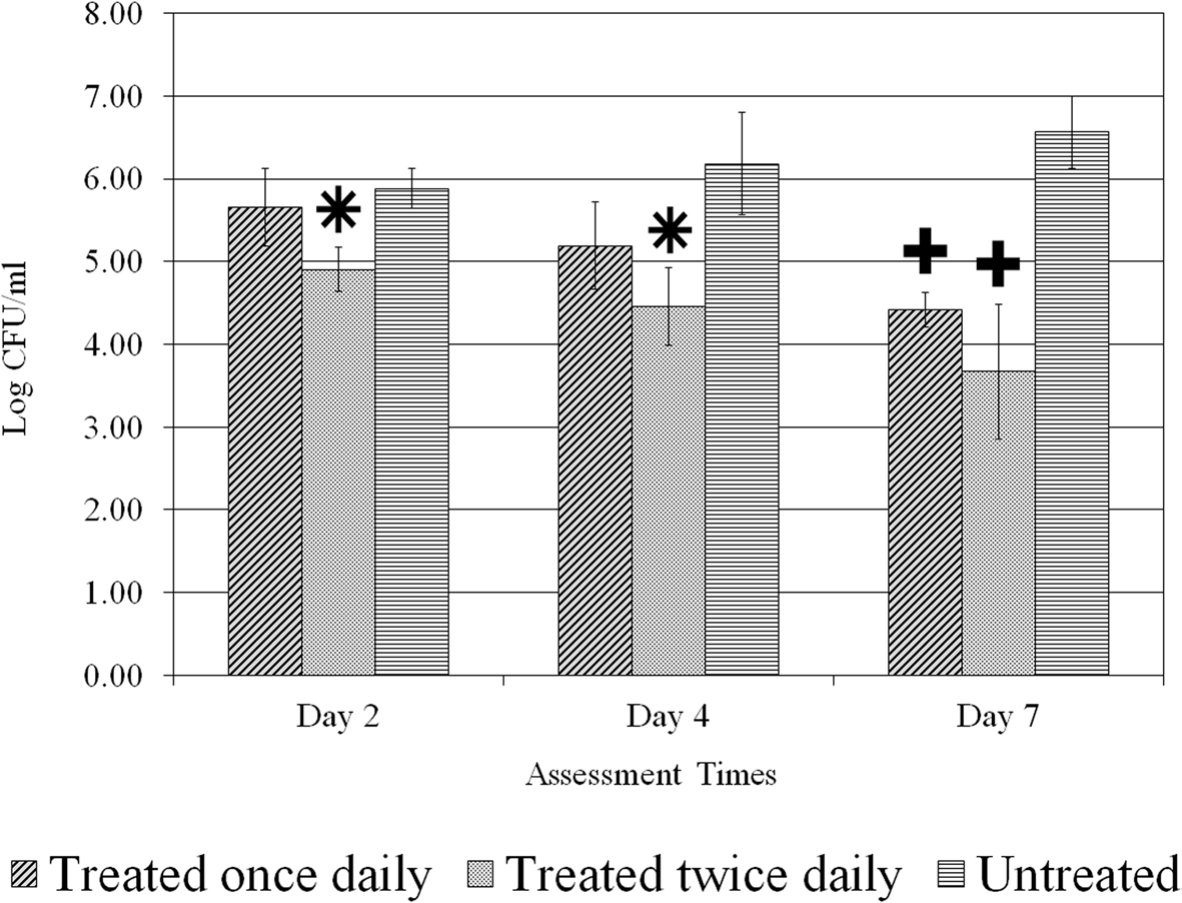
*C. albicans* (ATCC 10261) reference strain count. Comparison between different treatment regimens per assessment days. * *p* < 0.05 compared to other two treatment regimens,**+** *p* < 0.05 compared to Untreated

**Fig. 3:**
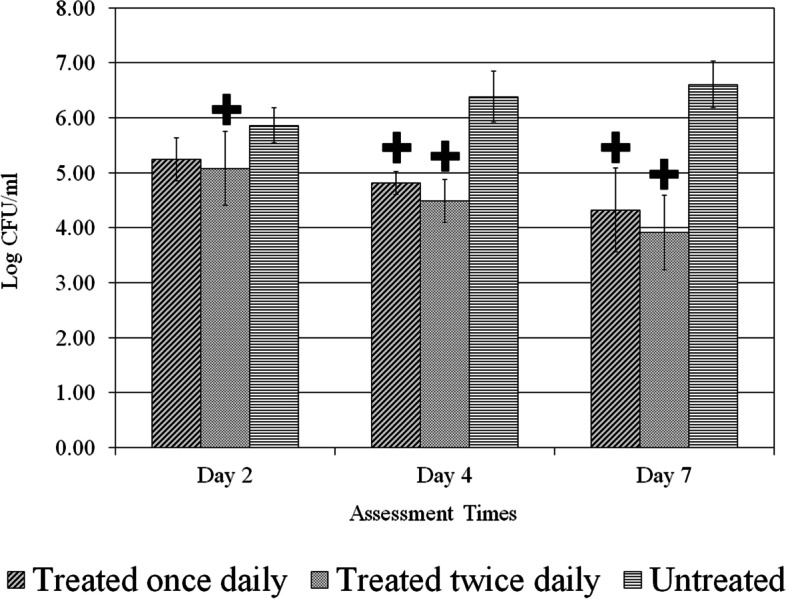
*C. albicans* (ATCC 64550) fluconazole-resistant count. Comparison between different treatment regimens per assessment days. **+** *p* < 0.05 compared to untreated

**Fig. 4:**
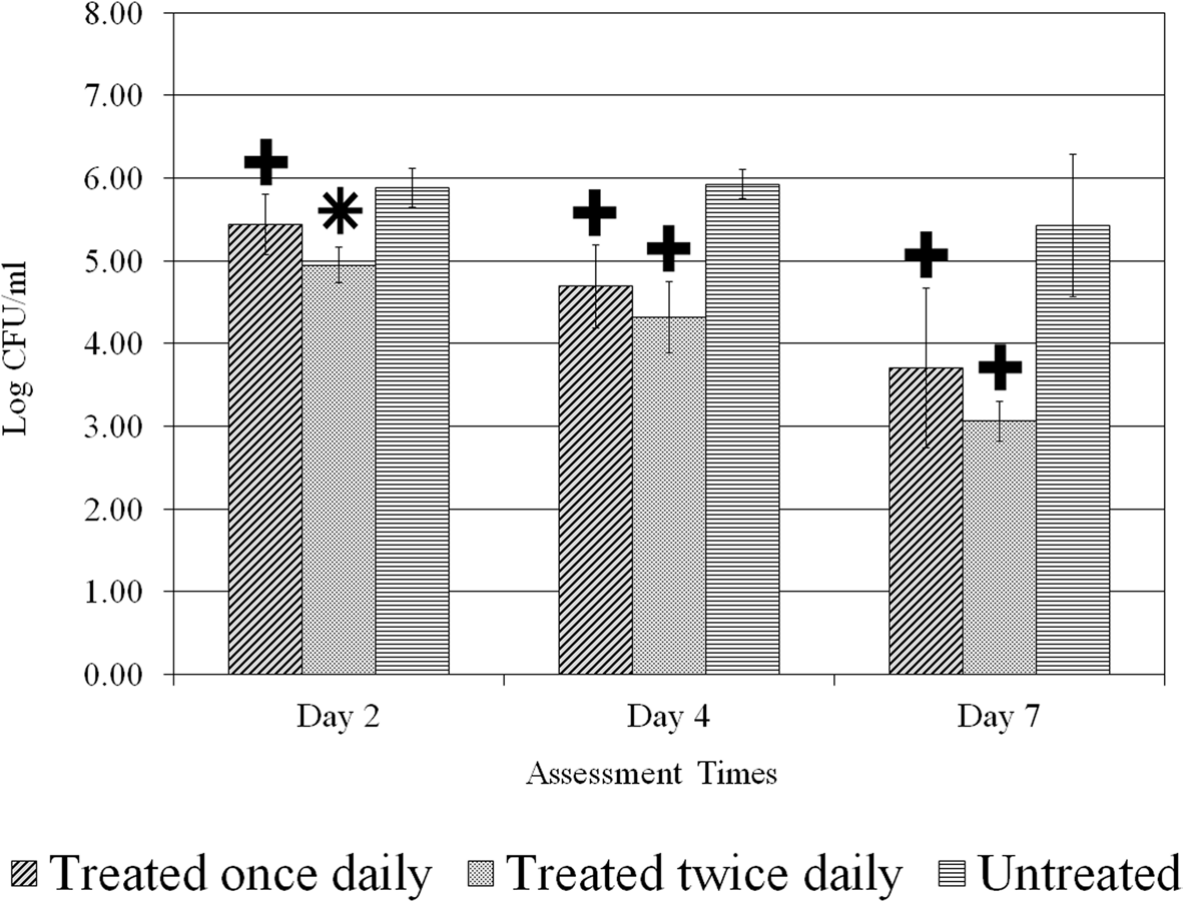
*C. albicans* (ATCC 26310) fluconazole-sensitive count. Comparison between different treatment regimens per assessment days. * *p* < 0.05 compared to other two treatment regimens, **+** *p* < 0.05 compared to untreated

A total of one hundred ninety-eight (198) wounds were evaluated. Treatment application for once or twice daily resulted in significant (*p* < 0.05) reductions for all *C. albicans* strains in comparison to the untreated control group as shown in Figs. 2, 3 and 4. The data also shows those wounds treated twice exhibiting lower fungal activity than those wounds treated once. The application of treatment twice daily resulted in a greater than 2.0 Log CFU/ml (*p* < 0.05) reduction of all strains of *C. albicans* compared to the untreated control on day 7 (Figs. 2, 3 and 4). For the reference strain *C. albicans* 10261 the twice daily treatment showed significantly (*p* < 0.05) better efficacy as compared to both once daily and untreated control on days 2 and 4 (Fig. 2). On day 7, treated twice daily wounds continued having significantly (*p* < 0.05) lower fungal counts when compared to those wounds left untreated, while remaining lower than those wounds treated once daily. Wounds infected with *C. albicans* 64550 (fluconazole resistant strain), on day 2 treated twice daily were significantly (*p* < 0.05) lower than those wounds left untreated a shown in Fig. 3. Both treatment regimens showed significantly (*p* < 0.05) lower fungal activity than untreated control (days 4 and 7). The counts for C. *albicans* 26310 (fluconazole sensitive) were significantly (*p* < 0.05) lower than untreated by both treatment regimens during all three assessment days (Fig. 4). On day 2, those wounds treated twice daily showed significantly (*p* < 0.05) lower counts than both treated once wounds and Untreated control. Those wounds treated twice daily on day 7 were substantially lower than those wounds left untreated, exhibiting the lowest fungal count for any of the three strains.

## Discussion

As shown in this study, the *in vivo* porcine model offers a useful model that can provide reliable translational data on wound infections on the epidermis and dermis layers. Swine skin structure is very similar to human skin, including having similar epithelial thickness of the stratum corneum [66]. Additionally, the swine model can provide a reliable model because of dermatophytes requiring keratin structures [67] found in said stratum corneum and pigs having a hair density comparable to human [68]. *Candida* species can be found as a normal flora in humans, commonly in infections related to hair and nails which can cause a systemic infection in the human body [69–71]. *Candida albicans* has been shown to be both sensitive and resistant to fluconazole [37, 38]. Polymicrobial communities is well known that could colonize chronic wounds, this colonization can delay the healing process [72, 73]. Recent studies showed that *Candida albicans* species could be found in a 22% of the isolations in chronic wounds [74]. Fungistatic characteristics of antifungal drugs, low doses of the drug, duration of the treatment as well interaction with other treatments are some of the reasons why limited treatment efficacy has been seen in patients with fungal infections as well for the resistance of different strains to the drugs [75]. Therapies such as azole fluconazole have been effective against most *Candida* species and are widely used as the first treatment option showed in an *in vitro* study to decrease the level of a Candidiasis [76]. In this *in vitro* study, Serpa et al. [73] tested a azole fluconazole treatment against *Candida albicans* ATCC 64550 fluconazole resistant showing a decrease in the counts after 7 days of treatments, cells were treated twice versus once, previous studies [50, 77] demonstrated that this strain have resistance to fluconazole treatments. Using *Candida albicans* ATCC 10261 as reference strain tested *in vitro* showed a low MIC when was challenged against fluconazole [78, 79]. In our study, *Candida albicans* ATCC 26310 fluconazole sensitive showed a decrease in the counts compared to untreated infected wounds however after 7 days the counts were higher to those wounds infected with the fluconazole resistance strain, others studies showed before that this strain decrease the counts after extended period of time more than 7 days [80].

Even when the wounds were treated twice for 6 days it was still insufficient amount to completely eradicate the fungal infection. Other authors demonstrated *in vitro* the combination of therapies as an effective methodology to reduce the candidiasis [81–83]. Cyclosporine in combination with fluconazole could be an effective treatment to complete eradication of fungal infection since increase the susceptibility to fluconazole due to efflux pump deletion or alteration of stress response caused by calcineurin during azole therapy [84].

Current literature has provided evidence for antifungal compounds to be active against other *C. albicans* strains. Pandolfi et al. [56] has reported evaluations of compounds having the same effects in different strains, with *C. albicans* ATCC 10261 showing a comparable biofilm activity as the other strains used in an *in vitro* study. Similarly, other *in vitro* studies challenging a range of 10–15 *C. albicans* strains have analyzed of other antifungal compounds not finding any collective difference in the results, such as a comparison including *C. albicans* ATCC 64550 [51].

We have developed a burn infection model which allows the colonization of *Candida sp* to examine the efficacy of topical and/or systemic antifungal therapies. One of the limitations with this study and all animal models used to assess the activity of antifungals is that they tend to be short‐term without underlying comorbidities, and do not necessarily replicate a true clinical infection, with clinical variables such as size, depth and aetiology [85–87].

## Conclusions

This study demonstrates the usefulness of a porcine second-degree burn model in evaluating treatment efficacy against *C. albicans* infections by developing a platform to compare different treatment groups against different strains. These results indicate that wounds treated with a topical terbinafine hydrochloride formulation had substantial reductions against *C. albicans* when comparing to untreated wounds. Despite the fungal presence not being fully eliminated on all wounds, the data in this study shows the significant reduction by treated twice wounds with terbinafine hydrochloride when compared to those wounds left untreated, thereby it can be extrapolated that the fungal bioburden can be eradicated with additional days.

Overall, we found that terbinafine treatments applied either once or twice a day exhibited lower reductions as the days progressed. Both treatment groups with terbinafine hydrochloride showed a desirable rate of reduction, which should further be investigated with additional assessment days or the *in tandem* application of another antifungal modality. This model will be beneficial to identify novel therapies that may be use clinically.

## Data Availability

The datasets generated and/or analyzed during the current study are not publicly available due raw data not being published for any other publication but are available from the corresponding author on reasonable request. The study was carried out in compliance with the ARRIVE guidelines.

## Abbreviations

ATCC: American type culture collection
MIC: Minimum inhibitory concentration
OTC: Over the counter
SPF: Specific pathogen free
CFU: Colony forming units
